# Pore Network Modeling of Intraparticle Transport Phenomena Accompanied by Chemical Reactions

**DOI:** 10.1021/acs.iecr.4c01727

**Published:** 2024-10-03

**Authors:** A. Fathiganjehlou, E. A. J. F. Peters, K. A. Buist, J. A. M. Kuipers

**Affiliations:** Multiphase Reactors Group, Department of Chemical Engineering and Chemistry, Eindhoven University of Technology, Postbus, 5600 MB Eindhoven, The Netherlands

## Abstract

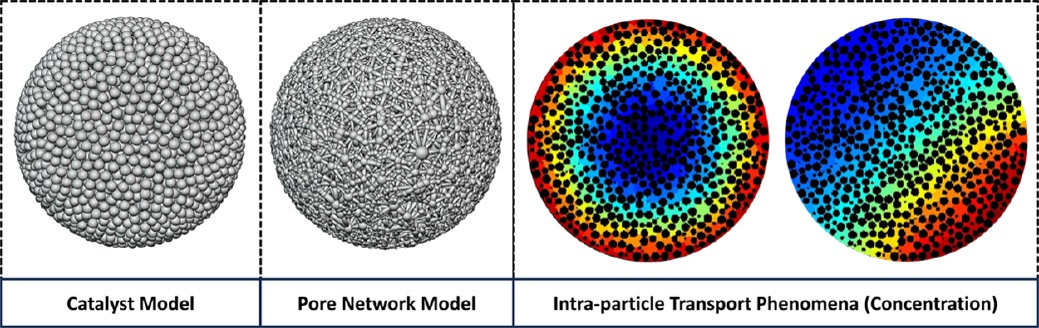

In this work, a 3D pore network model (PNM) is introduced for modeling reaction-diffusion phenomena, with and without coupled heat transfer, in a spherical porous catalyst particle. The particle geometry is generated by packing thousands of microspheres inside a large sphere to represent the 3D geometry, porosity, and tortuosity of a spherical catalyst particle. A pore-network representation is extracted from this geometry, and a PNM for diffusion-reaction and heat conduction is constructed. This newly proposed particle-scale PNM allows for the application of realistic 3D nonuniform boundary conditions on the particle’s surface, which is commonly encountered in slender packed-bed reactors. Concentration profiles inside the particle, and effectiveness of the reactions, is analyzed.

## 1 Introduction

The packed bed reactor is a chemical reactor with applications in various chemical processes such as adsorption, catalytic reactions, distillation, and separation processes. Some examples include oxidation processes such as the selective oxidation of ethylene^1^ and the oxidative coupling of methane,^2^ hydrogenation reactions such as CO_2_ hydrogenation,^3^ Fischer–Tropsch synthesis,^4,5^ Steam Methane Reforming (SMR),^6^ and many other chemical conversion processes.

In most packed bed reactors, chemical conversion processes involve either heat generation or heat consumption. Both exothermic and endothermic reactions are prevalent across various industries, petrochemical production, pharmaceuticals, materials processing, and energy generation. For example, the highly endothermic catalytic reforming of naphtha produces gasoline components, BTX aromatics (benzene, toluene, xylenes), and hydrogen in the refining and petrochemical industries.^7^ Additionally, steam cracking and catalytic cracking are endothermic processes, while hydrotreating and hydrocracking are exothermic.^8^ Multitubular packed bed reactors, characterized by a bundle of slender packed tubes with a low column-to-particle diameter ratio, are a favored reactor configuration for their effective heat management, particularly for highly exothermic reactions. These reactors facilitate efficient cooling by circulating a coolant between the parallel slender packed tubes.

At the core of these reactors are catalytic porous particles, where a complex interplay of intraparticle transport phenomena unfolds. The reaction predominantly occurs within the internal porous structure of the catalyst. The reactant diffuses into the pores of the particle and then reacts, a process governed by intraparticle transport phenomena. Understanding these phenomena is of great importance as they directly impact the performance and efficiency of the reactor.

In the case of exothermic reactions, the reaction generates heat during the reaction. The generated heat increases the rate constant of the reaction according to the Arrhenius law and consequently increases the reaction rate. The increased reaction rate generates even more heat and raises the temperature further. This interplay between the reaction rate and heat generation is called Arrhenius coupling, leading to unique behavior of the catalyst particle, such as the possibility of multiple steady states. Depending on the initial conditions, intraparticle transport phenomena inside a catalyst particle can result in two stable steady states: one ignited state and one extinguished state, along with one meta-stable point that represents a transient state.^9,10^

In the case of ignited steady states, elevated heat generation and reaction rates can lead to a local temperature increase within the packed bed. If the generated heat is not sufficiently removed, this localized temperature rise may result in the formation of hot spots and, subsequently, thermal runaway of the reactor.^11^ Flow mal-distribution, which is common in slender packed bed reactors,^12−14^ is one of the reasons for this insufficient heat removal. The significance of flow mal-distribution and partial-wetting on intraparticle transport phenomena and catalyst performance is emphasized in Harold et al.^15^ Therefore, a detailed understanding of intraparticle transport phenomena, especially in the case of nonuniform boundaries imposed on the particle, is crucial.

Several attempts have been made to investigate and model transport phenomena at the particle scale within a catalyst particle. Initial efforts primarily focused on mathematical and analytical approaches.^16^ One of the approaches developed for modeling intraparticle transport phenomena is based on the volume-averaging theorem.^17−23^

Volume-averaged continuum models offer a means to express essential transport and reaction parameters, such as effective diffusivities and reaction rates, based on measurable macroscopic quantities. In these models, the physical properties of the entire porous medium are represented using equivalent averaged quantities. While these models are computationally efficient, complex porous media often necessitate experiments to determine equivalent properties, which can be challenging, especially in complex multiphysics systems such as coupled reactions, diffusion, and heat transport. Additionally, the averaging of properties within the porous medium results in the loss of local information, which can pose challenges in complex porous media, such as highly anisotropic materials.^24^

In recent years, advancements in computational methods and hardware have enabled the investigation of transport phenomena for realistic geometries of porous materials using pore-scale models. In these models, intraparticle transport phenomena are analyzed at the pore scale within the void space of the porous medium, eliminating the need for measuring equivalent properties, as is required in continuum models. Utilizing readily available bulk fluid properties from existing literature suffices for pore-scale models. One such computational technique, Computational Fluid Dynamics (CFD), includes methods such as Direct Numerical Simulation (DNS), which operate at a fine spatial (microscale) and temporal resolution, providing detailed, locally resolved information while accounting for realistic geometrical heterogeneities. However, it is worth noting that microscale methods come with the drawback of being computationally expensive.^25−27^

Pore Network Model (PNM) represents another pore-scale approach for modeling intraparticle transport phenomena. PNM operates at a meso scale, where the intricate porous geometry is simplified into a network comprising pore bodies and throats. This network retains essential information about pore geometry, interconnectivity, and tortuosity.^28^ Given the relatively small length scale of the pores, it is reasonable to assume that variations in properties such as concentration and temperature are negligible within a pore due to the short mixing time scale.^24^ The simplification of the geometry increases the length scale of the system, roughly one or two orders of magnitude larger than that of microscale methods, significantly reducing computational demands while still providing locally resolved information. In essence, PNM serves as a bridge between microscale and volume-averaged models, combining the advantages of both approaches.

In recent years, PNM has been employed to model hydrodynamics and transport phenomena in packed bed and trickle bed reactors, with a focus on modeling the phenomena on the reactor scale.^13,29−35^ Furthermore, various efforts have been made to apply PNM for modeling intraparticle transport phenomena on the particle scale.^24,36−40^ However, the previous studies on modeling intraparticle transport phenomena using PNM have treated PNM as a simple-cubic or structured network of interconnected pores and throats.^41−46^ Jerauld et al.^47^ and Winterfeld et al.^48^ have shown that as long as the average coordination number of a topologically disordered system is equal to the coordination number of a regular network, the transport properties of the two systems are essentially identical. This is the basis for the use of simple cubic pore networks. In a study by Ye et al.,^49^ a method was developed for generating regular and irregular pore networks in porous particles of arbitrary shape, with a particular focus on its application to the catalytic hydrogenation of benzene. Additional research in the field includes multiscale Boolean modeling of platelets to replicate the porous structure in γ-Alumina supports.^50,51^

In this research, we construct a spherical agglomerate composed of ten thousand randomly positioned nonoverlapping microspheres to mimic the 3D geometry of a spherical catalyst particle. From this catalyst model, we extract the 3D particle-scale pore network and define surface pores and throats to impose boundary conditions on the particle-scale PNM. This definition of surface pores and throats allows us to apply 3D nonuniform boundary conditions, which are common in slender packed bed reactors.^12^

In the particle-scale PNM, diffusion resistance is attributed to the throats. Analytical expressions for this resistance are readily available when assuming simple geometries for the throats, such as straight tubes with circular cross sections. However, in reality, throats exhibit more complex shapes, and over-simplification can result in less accurate results. To enhance the precision of PNM, calibration is necessary. In the literature, various methods have been explored to address this issue, including PNM calibration using experiments,^29^ empirical correlations,^30^ and particle-resolved numerical methods.^52,53,34^ In the present study, calibration is conducted by fitting the steady-state effectiveness factor, for each particle at different Thiele-modulus, to the analytical effectiveness factor for steady-state diffusion and reaction inside a homogeneous spherical particle.^9^ The calibration parameter in PNM is the scaling factor for the throat radius, *F*.

The optimized parameters are utilized to implement intraparticle transport phenomena in the developed PNM. Initially, a reaction and diffusion system is assumed within the PNM, and the average steady-state radial concentration profiles are compared with analytical solutions. Subsequently, the model is extended to include reaction-diffusion coupled with heat transport, a novel element compared to existing literature. The validity of PNM in modeling this coupled phenomenon is assessed by comparing values of the effectiveness factor to the numerical solution proposed by Weisz and Hicks.^9^ Furthermore, the capability of PNM to capture multiple steady states resulting from the Arrhenius coupling is investigated. Finally, several examples demonstrate the capability to accommodate nonuniform 3D boundary conditions in the developed PNM which is prevalent in for instance trickle bed reactors.

In summary, our work presents several novel contributions. First, we introduce an efficient particle-scale PNM capable of simulating reaction-diffusion systems with and without heat transport. This PNM is notable for its ability to provide partially resolved local results in a matter of seconds. While in other methods such as CFD, this implementation takes much longer.^54,55,25^ The exact same modeling framework can be used to model the particles in the packed bed, enabling efficient and fast modeling of large-scale systems with many particles. Additionally, we incorporate a 3D realistic geometry of catalyst particles into our model, allowing for the application of geometric treatments such as imposing nonuniform boundary conditions. Additionally, while our focus primarily lies on spherical particles in this work, the framework is flexible and can be extended to accommodate nonspherical particles in future studies. Furthermore, our work marks the successful implementation of heat transport phenomena using PNM, effectively capturing coupled heat-reaction effects such as multiple steady states and local heat generation, which, to the best of our knowledge, has not been addressed using PNM before.

## 2 Methods

### 2.1 Model Geometry

**Figure 1:**
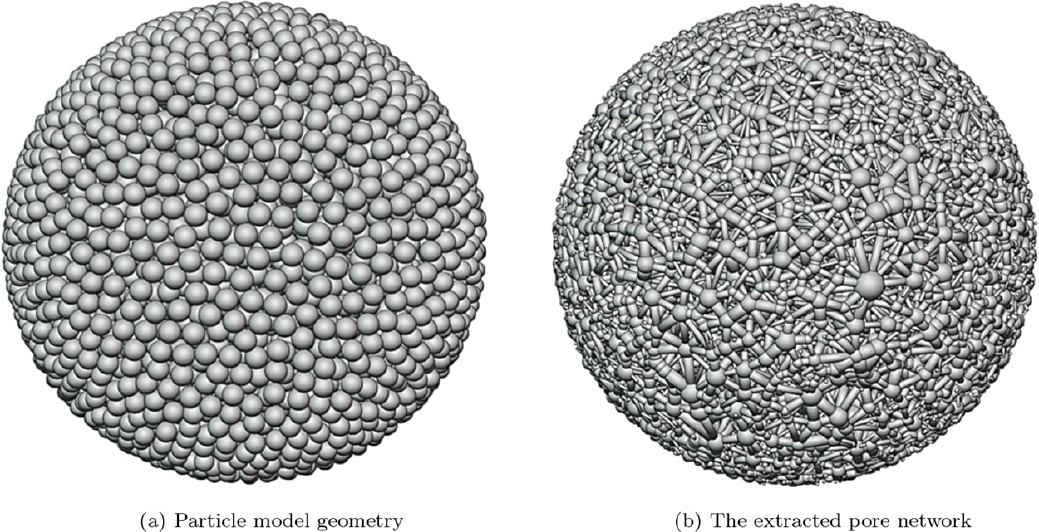
(a) Particle model geometry consisting of an agglomeration of 10,000 randomly positioned microspheres with a uniform size distribution. (b) The corresponding extracted pore network.

A conglomerate of nonoverlapping microspheres, arranged randomly, is created to mimic the porosity and tortuosity inherent to the porous catalyst particle. To create a random close packing into the placement of spheres, we utilize the Lubachevsky-Stillinger algorithm.^56^ Within this algorithm, a predetermined number of points is randomly positioned within a larger sphere, representing the outer boundary of the macroscopic catalyst particle. Following their initial placement, the dimensions of the spheres undergo incremental enlargement at a defined growth rate until either two spheres come into contact or a sphere touches the boundary of the pellet. Subsequently, the involved spheres undergo adjustment through an elastic collision mechanism. The use of an event-driven approach ensures the prevention of sphere overlap. When implementing this algorithm, it is crucial to select a sufficiently high growth rate to immobilize the spheres in their positions, thereby preventing the formation of an undesired crystalline lattice. For more detailed information, please refer to ref (25). In this study, we consider a 3D model of a spherical particle with 1 mm diameter, composed of ten thousand microspheres with a radius of 18.42 μm centered within the particle (Figure 1a).

### 2.2 Pore Network Model

The Pore Network Model (PNM) is employed to simulate intraparticle transport phenomena within the catalyst particle. In PNM, the complex 3D void space is represented as pore bodies and throats, which capture the void space and its interconnectivity. The PNM is reconstructed from post-processed 3D structural images of the particle. This 3D volume image is voxelized into 1024 × 1024 × 1024 voxels (volume elements) with a voxel size of 0.9765 μm. Then, the voxelized image is binarized with a value of 1 representing the solid, and the voxels with a value of 0 representing the void space. Voxelization and binarization are performed using the Slicer software.^57^ The pore network is then extracted from this binarized image using a maximal ball pore network extraction method.^53,58,59^Figure 1b illustrates the extracted PNM from the model catalyst particle.

It is important to note that, after extracting the pore network, isolated pores, that have no connections to other pores, are observed. The sum of the volumes of these isolated pores is negligible compared to the total pore volume in the particle (≈ 0.16%), so they are eliminated from the PNM.

### 2.3 Reaction and Diffusion

Combined diffusion and reaction is implemented on the extracted PNM. The particle is assumed to be submerged in a bath with a non-dimensional concentration equal to 1. Solute molecules diffuse through the porous space of the particle in the radial direction while simultaneously undergoing a reaction. It is assumed that the reaction exclusively takes places in the pores. Throats are assumed to facilitate only diffusion, with no diffusion occurring in the perfectly mixed pores. For diffusion, Fick’s law is applied, and it is assumed that solute diffusion occurs solely through the throats. The molar flow rate, *n*_α_, through a throat, α, is governed by Fick’s law. This is mathematically represented as
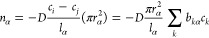
1where, *c*_*i*_ and *c*_*j*_ represent the concentrations in two interconnected pores, namely *i* and *j*, linked by the throat α. The throat length is *l*_α_, and *r*_α_ is its radius, and vary for each throat. The diffusion coefficient is denoted by *D*. Eq 1 could be extended by a multicomponent Stefan-Maxwell approach.

Fick’s law dictates that the molar flux is proportional to the concentration gradient, here represented as the difference between *c*_*i*_ and *c*_*j*_ divided by *l*_α_. By multiplying this flux by the cross-sectional area of the throat, given as π*r*_α_^2^, we obtain the molar flow rate, *n*_α_. This derivation assumes a linear concentration gradient along a straight, cylindrical throat.

However, real throat geometries often deviate from this idealized shape. The throat radius is derived from the pore radius provided by the PNM extraction method. The direct equivalence of this radius to *r*_α_ is not inherently evident. Therefore, *r*_α_ is frequently treated as an adjustable parameter within the PNM to better match the obtained transport rate with empirical information. Further discussion on this calibration will follow.

The second part of eq 1 introduces an incidence matrix with elements *b*_*k*α_. These elements define the connectivity of pores and throats within the network. For instance, if a pore *i* is connected to another pore *j* via throat α, the matrix values are set as *b*_*i*α_ = −1, *b*_*j*α_ = 1, and *b*_*k*α_ = 0 for all other *k* that do not equal *i* or *j*.

This matrix not only indicates connectivity but also incorporates directional information through its sign convention. In the example, the negative value at *b*_*i*α_ and the positive value at *b*_*j*α_ imply that the throat begins at pore *i* and ends at pore *j*. Consequently, a positive *n*_α_ indicates molar flow from *i* into *j*, while a negative value signifies flow in the opposite direction, from *j* to *i*.

For each pore, with volume *V*_*i*_, we can write a balance equation, where accumulation is balanced by transport into the pore and reactions. Using the incidence matrix introduced above, the total molar flow entering pore *i* from all throats connected to it can be conveniently summed as ∑_α_*b*_*i*α_*n*_α_. The mole balance for a species with concentration *c*_*i*_ in pore *i*, in case of a first order volumetric reaction, is
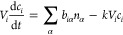
2We describe the reaction as volumetric inside the pore bodies. It can be argued that catalytic reactions are surface reactions and therefore *k*_s_*S*_*i*_, with *k*_s_ a surface reaction rate and *S*_*i*_ the surface area corresponding to pore *i* should be used. For reasons of convenience we did not implement the surface reaction. However, due to the small length-scale of the pores and the resulting negligible variations in concentration within individual pores, the assumption of volumetric reactions remains reasonable.^24^ Nevertheless, surface reactions can be included in the PNM by defining surface fragments. In our previous works, we used the definition of surface fragments to determine the local particle wetting of particles in a trickle bed^14^ and also to couple the particle-scale PNM to the reactor-scale PNM.^60^ By dividing each microsphere’s surface into a number of surface fragments and locating the center of each surface fragment in the volume element map, we can calculate the surface area of the pores in contact with the microspheres. Using this information, surface reactions can be implemented in the PNM. However, for reasons of convenience, we did not implement the surface reaction.

#### 2.3.1 Boundary Conditions

**Figure 2:**
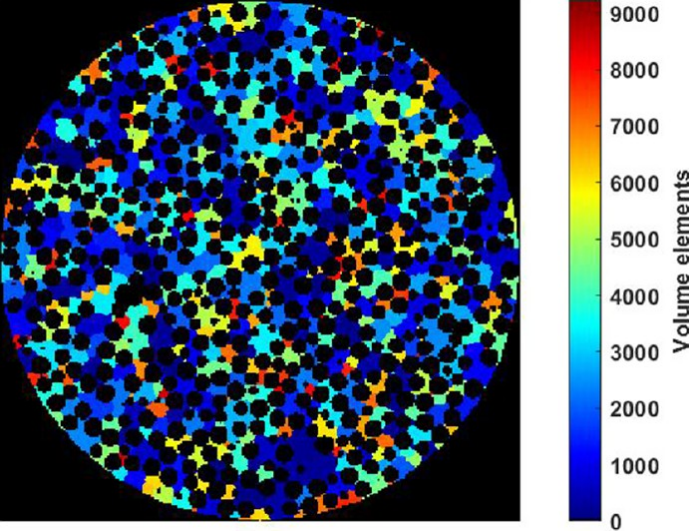
A schematic of the volume elements map. Each volume element correspond to a pore body in the PNM. The 3D volume element map is imported, postprocessed and visualized in Matlab.^61^

In the developed PNM, the boundary condition on the particle’s surface is imposed by introducing inlet throats. This process begins with the definition of surface pores, which represent the pore bodies located on the particle’s surface. To identify these surface pores, the volume element map is utilized. Figure 2 illustrates the volume element map for the particle. The volume element map is generated during the pore network extraction, with each volume element corresponding to a pore body within the PNM. To identify the surface pores, a spherical shell with a diameter of 1 mm and a thickness equivalent to four voxels is assumed. The volume elements that contain a voxel within this spherical shell are considered as the surface pores.

For each surface pore, we introduce an inlet throat that links the surface pore to the outer surface of the pellet, where the concentration is set to 1. The radius and length of the surface throat are taken as equal to the radius of the corresponding surface-pore. This connects a point on the particle surface to a surface-pore.
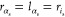
3where *i*_s_ labels the surface-pore that is connected to the throat (i.e., *b*_*i*_s_α_s__ = 1). The concentration used at the surface, *c*_s_ is imposed on the outer side of the surface throat.

As discussed, we assume a linear concentration profile along the throat length to derive the expression for the molar flow rate through a throat, eq 1. This is not appropriate when reaction rates are high. In that case, a significant amount of material can react already when being transported through a throat. If reaction rates are so high, a significant amount of material will therefore be consumed in the surface throats.

To correct for this case, we use an expression derived from the stationary profile in a surface throat of length *l*_α_s__ including reaction. The stationary profile follows from
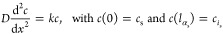
4with analytical solution:
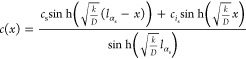
5According to this expression, the flux at the surface (*x* = 0) equals
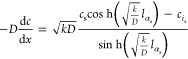
6Note that for *k* small we recover, Fick’s law, *D* (*c*_s_ – *c*_*i*,s_)/*l*_α_s__, while for large reaction rates we find a flux corresponding to reaction-diffusion in an infinite slab. The crossover depends on the throat’s Thiele modulus .

### 2.4 Numerical Solution Method

In our implementation, we use an Euler-backward discretization of the time evolution. The time discretized form of eq 2 is
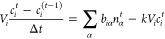
7From this equation the concentration at the current time index, *t*, can be computed from the one at previous time index *t* – 1. However, the to-be-computed concentrations, *c*_*i*_^*t*^ also appear on the right-hand-side. Note that also the molar flows linearly depend on the concentrations by means of eq 1. Therefore, a set of linear equations is formed. This set can be best written in the form of a matrix-vector equation and solved using a standard linear solver.

In this matrix-vector equation, the concentration in the pores, *c*_*i*_, are listed in a column vector: **c**. The incidence matrix is written as **B** with elements *b*_*i*α_, as introduced before. Note that this matrix is very sparse, as are also the other matrices that will appear. Therefore, in the implementation, a sparse-matrix storage format and sparse-matrix solver should be used.

Using Fick’s law, we can calculate the molar flows, represented as **n**, through the throats using the following equation:
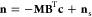
8where, **M** represents the diffusion conductance matrix, which is a diagonal matrix containing the diffusion conductance per throat. For nonsurface throats this matrix has elements:
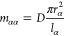
9

For surface throats, there is a contribution to the molar flow due to the imposed concentration at the surface, *c*_s_. This gives nonzero entries into the vector **n**_s_ and custom values for entries in **M**. For a surface throat, α_s_, the diffusion conductance matrix can be determined from eq 6 as
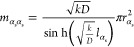
10With the choice of positive sign of the incidence matrix entry for the inlet pore *i*_s_ (i.e., *b*_*i*_s_α_s__ = 1), the inhomogeneous contribution to the molar flow is
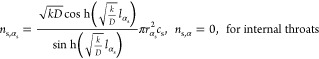
11

The matrix-form of eq 7, including the implementations of the boundary conditions, is
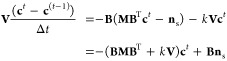
12where, **V** is a diagonal matrix that contains the volume of each pore, Δ*t* is the time step, **c**^*t*^ is the concentration vector at time index *t*, and **c**^*t*–1^ is the concentration vector in the previous time step.

Collecting of all terms proportional with **c**^*t*^ on the left-hand-side, and all others to the right gives the matrix-vector equation that needs to be solved for each time step:
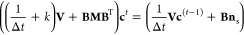
13

In our Matlab implementation, we used the standard backslash operator to solve the matrix-vector equation. This invokes a direct sparse solver. For large systems, one might consider some optimization. In the case of a first order reaction, as considered here, the matrix does not change in time. So, one can perform the needed matrix-factorization once and reuse it each time step. Beyond a certain problem size, it should become more efficient to use an iterative solver, such as the preconditioned conjugate-gradient method. In principle the solution presented in this section for first-order equations can be extended to systems of nonlinear kinetic equations. The pore model remains unaffected.

For other kinetic orders or reversible reactions, iterative numerical methods would need to be incorporated into the model to handle the nonlinearity and potential equilibrium states. For instance, with Langmuir–Hinshelwood kinetics, which involve adsorption and surface reactions, the model would need to include additional terms to account for these processes. This would likely involve solving a set of coupled nonlinear equations to describe the interaction between the adsorbed species and the reaction rates.

### 2.5 Calibration

In the particle-scale PNM, diffusion resistance is assigned to the throats, which are idealized as straight tubes with circular cross sections (*s*_α_ = π*r*_α_^2^). Nevertheless, real throats exhibit more intricate geometries, including variations in cross-sectional area along their length. This over-simplification can lead to less precise predictions, as the interplay between *s*_α_ and *l*_α_ introduces the effects of porosity and tortuosity on the effective flux. To address this simplification, a throat radius correction factor (*F*) is introduced:
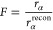
14where, *r*_α_^recon^ refers to the cylindrical throat radius obtained from the maximal ball algorithm, and it is essentially the radius of the smallest cylindrical ball that can traverse the path between pores *i* and *j*. The throat radius correction factor (*F*) will serve as the optimization factor to calibrate the PNM.

The calibration is carried out by fitting steady state results of the PNM with an analytical solution for the diffusion-reaction in a homogeneous sphere with first order reaction. The governing equation for the spherical symmetric diffusion is
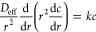
15where, *D*_eff_ is an effective diffusion coefficient taking into account the porosity and tortuosity of the porous material, and *k* is the first-order reaction constant. The ratio of the reaction rate to the diffusion rate is characterized by a dimensionless number known as the Thiele modulus. The ratio of the particle radius compared to a typical distance the species diffuse before reacting away is the Thiele modulus φ,
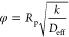
16For large Thiele modulus the concentration profile will decay steeply when moving further inside the particle, and the effective reaction rate inside the particle will deviate significantly from that at the boundary.

The effectiveness factor, η, is defined as the ratio of the actual reaction rate within the catalyst particle to the hypothetical reaction rate when the entire particle is assumed to be immersed in the surface reactant concentration. For the 1D spherical symmetric problem of eq 15 the effectiveness factor equals,
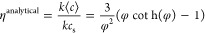
17In the PNM the effectiveness factor can be computed as
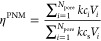
18

The calibration of the PNM is carried out by fitting the PNM steady-state effectiveness factor to the analytical expression for a homogeneous spherical particle, eq 17, at various values of the Thiele modulus. However, the Thiele modulus is defined based on the effective diffusion coefficient, *D*_eff_, while in PNM a local diffusion coefficient is used. Several attempts have been made to determine the relation between *D*_eff_ and *D* in porous media.^62,63^ A correlation, which is commonly applied to homogeneous random spherical packings, is^64^
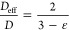
19ε is the mean porosity of the particle, which is
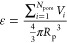
20To achieve a more accurate correlation for *D*_eff_ and effectively incorporate the effects of porosity and tortuosity, precise experiments and/or detailed microscale simulations, such as PR-CFD, are necessary.

**Figure 3:**
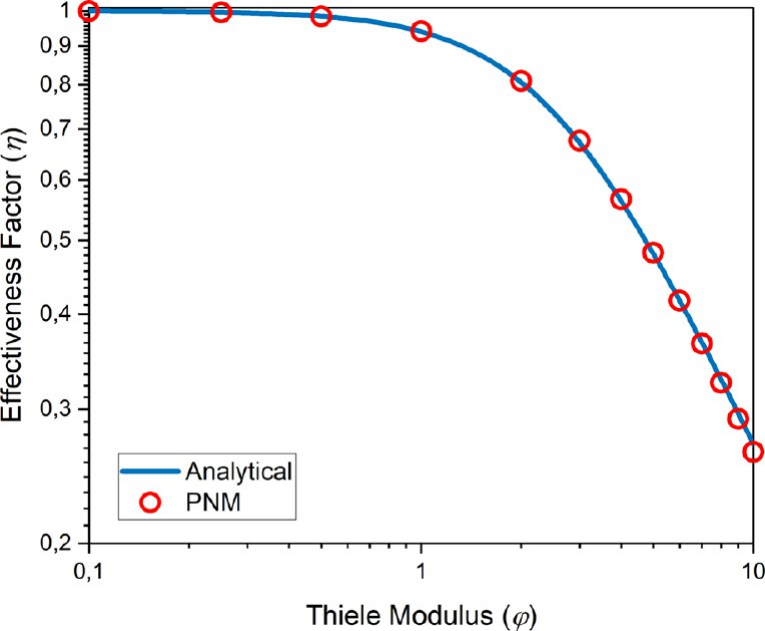
Effectiveness factor (η) curve for various φ values.

The steady-state effectiveness factor is calculated using the PNM for various φ values and is then fitted to the analytical effectiveness factor through a multi-variable search optimization approach (Figure 3). The Genetic Algorithm (GA) of the optimization toolbox v8.3 of Matlab 2019a^65^ is used for this optimization process. The objective function used in the optimization is the root-mean-square (rms) of the difference between the effectiveness values obtained from the PNM and the numerical solution. The optimization parameter is the scaling factor for the throat radius, *F*. The optimization process has converged with an initial population of 25 and a maximum of 25 generations. An optimal value of  is obtained with an rms = 0.30%. Figure 3 shows the values of η^PNM^ and η^analytical^ plotted against φ.

**Figure 4:**
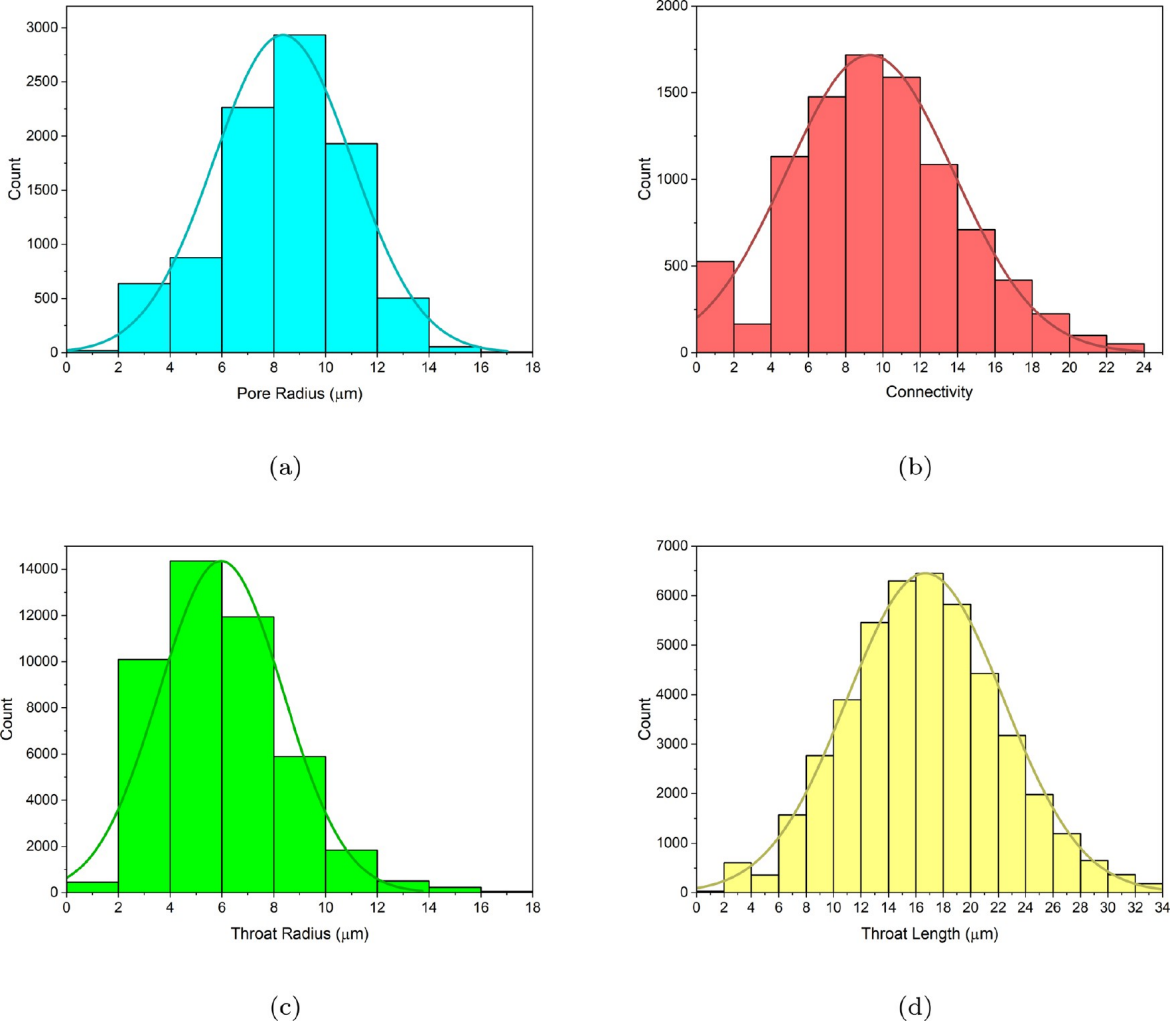
Distribution of (a) pore radius, (b) pore connectivity, (c) throat radius, and (d) throat length in the pore network.

Table 1 provides a summary of the final pore network used in the remainder of the manuscript, and Figure 4 illustrates the distribution of pore radius, pore connectivity, throat radius, and throat length within the pore network.

**Table 1:** Properties of the Final Pore Network Used in the Remainder of the Manuscript

pore network properties	value
porosity (ε)	0.493
total pore volume	0.258 mm^3^
total throat surface area	5.891 mm^2^
average connectivity	9.3
number of pores (*N*_pore_)	9231
number of throats (*N*_thr_)	45381

### 2.6 Reaction, Diffusion, and Heat Transport

In this section, the diffusion-reaction inside the porous particle is coupled with heat transport. The heat generated by the reaction raises the temperature inside the pore, subsequently influencing the reaction rate constant (*k*) according to the Arrhenius equation:
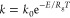
21where, *E* is the activation energy of the reaction, *R*_g_ is the universal gas constant, and *k*_0_ is the Arrhenius pre-exponential factor. The reaction constant *k*, in turn, influences the heat generated by the reaction, thereby affecting the temperature profile. This underscores the nonlinear interplay between the concentration and temperature profiles within the particle.

To incorporate the diffusion-reaction coupled with heat transfer physics into the PNM, we also solve both a mole and heat balance for each pore. For example, for pore *i*, these equations become:
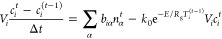
22
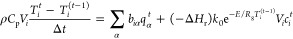
23The mole balance is essentially the same as before in eq 7, with the only difference of the Arrhenius coupling in the reaction term. The heat balance is very analogous to the mole balance. Differences are some extra factors, such as the heat-capacity, the accumulation term, and reaction enthalpy in the source term. Instead of the molar flow that follows from Fick’s law, eq 24, here we have a heat flow, *q*_α_, for each throat that obeys Fourier’s law:
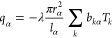
24

**Figure 5:**
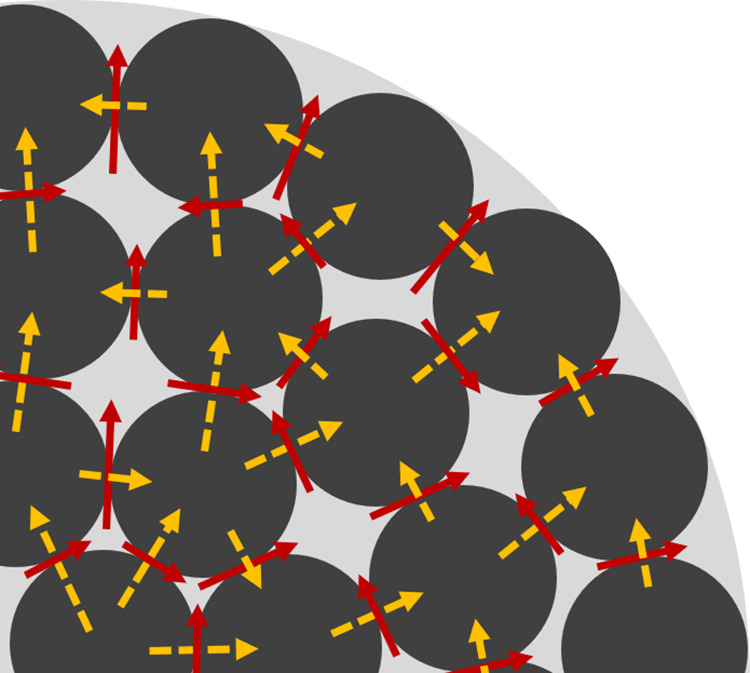
A schematic of a section of the particle is presented, with dark gray circles representing the microspheres and the light gray area depicting the void space. The actual conduction pathways through the microspheres are plotted in dashed yellow arrays. Meanwhile, the red arrows represent the heat pathway modeled in the PNM.

It is important to note that for the sake of ease of calculations, we modeled the conduction via the throats that connect the voids, while in reality conduction through the solid phase will dominate. The contribution of the solid phase is, however, incorporated in the value of the thermal conductivity, λ. Because the thermal diffusivity is much higher than the mass diffusivity, temperature gradients will be less steep than concentration gradients. Therefore, results will not heavily depend on details of the network that was used to solve the heat balance. Thus, it is possible and convenient to use the pore-network to describe the heat transport. Figure 5 depicts a schematic of the heat pathways for conduction within the particle. This figure illustrates a section of the particle, with dark gray circles representing the microspheres and the light gray area depicting the void space. The conduction pathways through the microspheres are delineated by dashed yellow lines, while the red arrows represent the heat pathway modeled in the PNM. A thermal conductivity value of λ = 0.493 W/(m · K) is used for the heat conduction coefficient of the particle.

Numerically, the temperature is evaluated implicitly in most terms except for the Arrhenius term where it is evaluated explicitly. This has as benefit that the set of equations that need to be solved, each time step, for the concentrations and temperature are decoupled and remain linear. When solving the mole balances first, this gives concentrations, *c*_*i*_^*t*^, that is needed for the reaction term in the heat balance. In matrix-vector form, the equations are
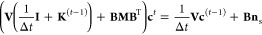
25
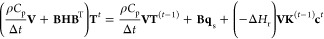
26where the matrix **K** is a diagonal matrix that contains the reaction coefficient, eq 21, for each pore. This matrix changes in time, because it depends on the temperature evaluated in the previous time step, i.e., *T*_*i*_^(*t*–1)^. The matrix **H** represents the heat conductance matrix, which is a diagonal matrix containing the heat conductance per throat.
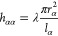
27Differently, from the diffusion case we also use this expression for the surface throats, because temperature profiles are expected to be much flatter than concentration profiles. The vector **q**_s_ contains the contribution to the heat-flows of the temperature imposed at the surface, *T*_s_,
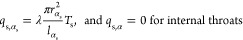
28

## 3 Results and Discussion

Utilizing the optimized value for *F*, the analysis of reaction-diffusion with and without heat transport is conducted using the PNM. The PNM simulations are performed on a computer equipped with an AMD Ryzen Threadripper 3970X 32-Core Processor (3.70 GHz) and 256 GB of RAM. For the reaction and diffusion case, the actual CPU time is a fraction of a second. For the reaction and diffusion coupled with heat transfer, depending on the values of φ and β, the simulation time ranges from a fraction of a second to a couple of seconds.

### 3.1 Reaction and Diffusion

The steady-state analytical solution of eq 15 inside a homogeneous spherical particle yields the following expression for the radial concentration profile^9^:
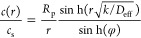
29

**Figure 6:**
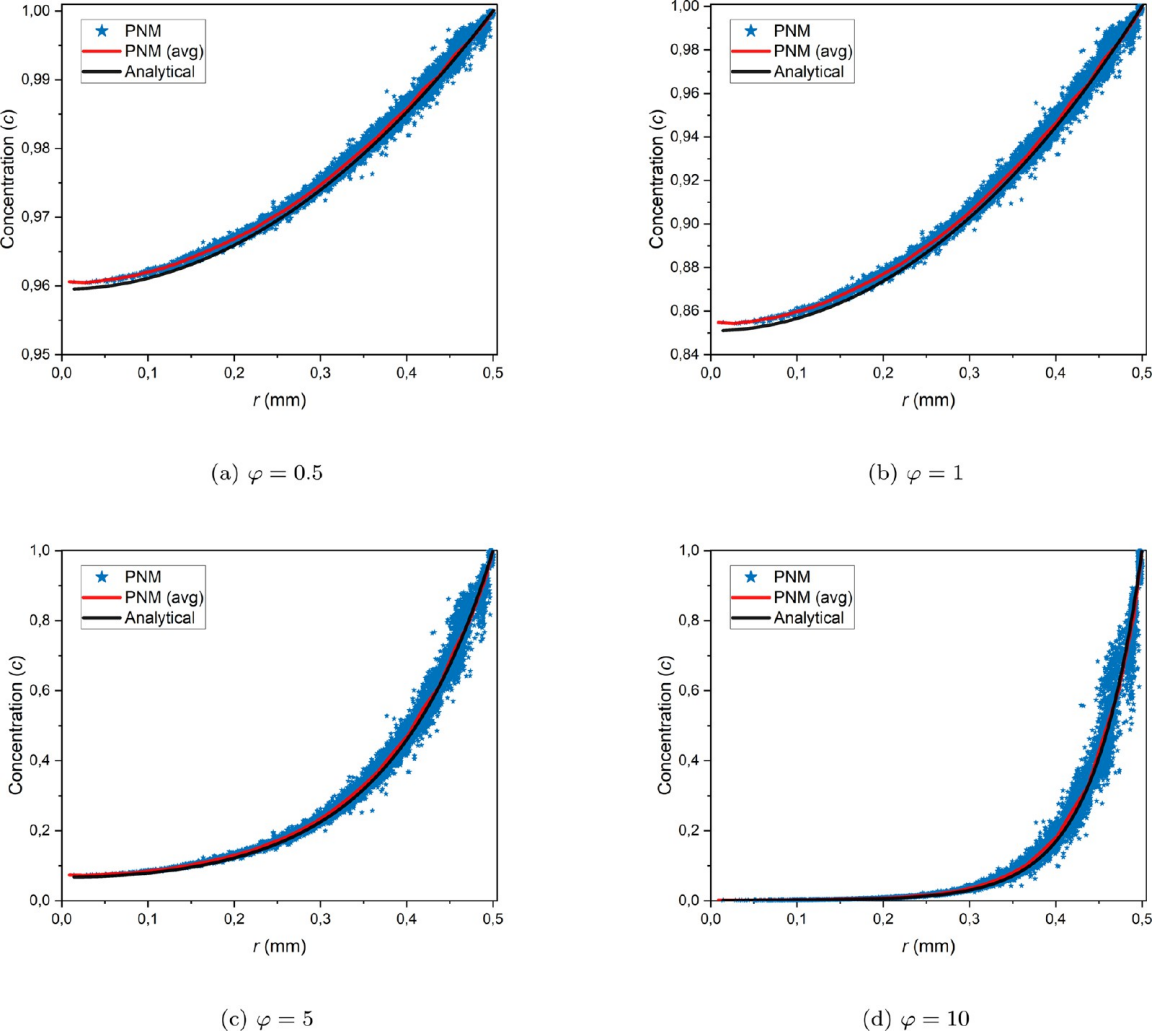
Radial steady state concentration profiles from PNM and the analytical solution.

To obtain the radial concentration profile from PNM, the pore bodies are sorted in the radial direction, and the pore concentration values are plotted for four different φ values and compared with the analytical radial concentration profile in Figure 6. The average pore concentration profile (plotted in red), calculated by binning in the radial direction and averaging the pore concentration values within each radial bin, corresponds well with the radial concentration profile predicted by the analytical solution. However, there is a slightly weaker correspondence for lower φ values, particularly near the particle center. This is due to the lower number of pores and throats near the center, resulting in a less-resolved system.

**Figure 7:**
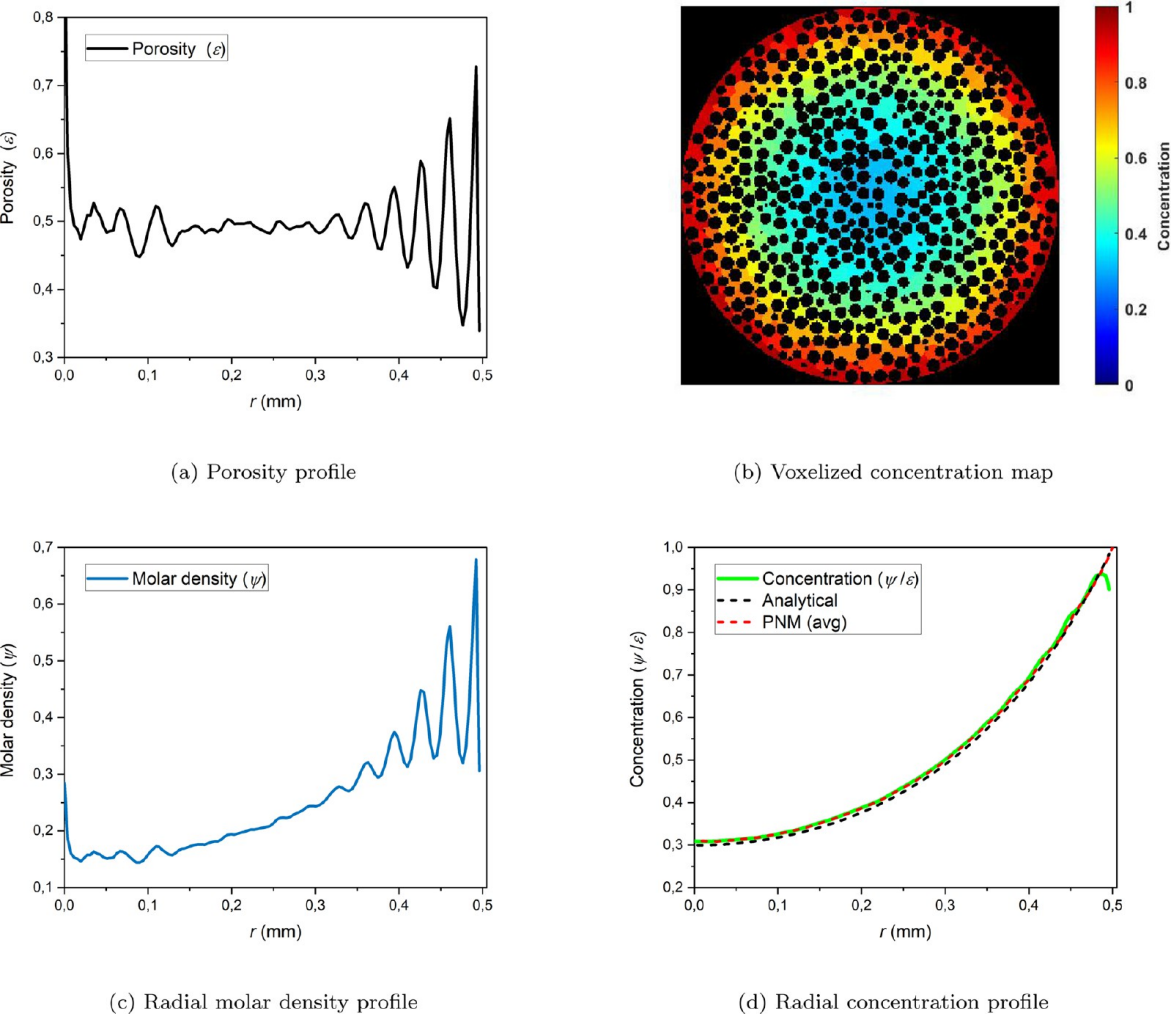
(a) Radial porosity profile, (b) voxelized concentration map from the middle slice of the particle, (c) radial molar density profile, and (d) radial voxelized concentration profile (colored in green) and average pore concentration profile (colored in red) obtained from PNM and radial concentration profile from the analytical solution (colored in black) at φ = 3 at steady state.

In Figure 6, the perturbations in the radial concentration profile from PNM are primarily attributed to the particle geometry and the arrangement of constituent microspheres. To delve deeper into this phenomenon, we have plotted the radial porosity profile of the particle geometry (Figure 7a). The porosity profile is computed based on the binarized 3D image. The particle radius is segmented into 128 rings, and within each ring, the sum of voxels with a value of 0 (void voxels) is divided by the total number of voxels in that specific ring.

In the PNM, we calculate the concentration value in each pore. These pore concentration values are then mapped to volume elements using a volume element map, which involves assigning a concentration value to each voxel based on the corresponding pore concentration. This resulting 3D map is referred to as the ‘voxelized concentration map’. Figure 7b displays the voxelized steady-state concentration map in the middle slice of the particle at φ = 3.

In Figure 7c, the radial molar density profile is presented. This profile shows the amount of moles per volume at each radial ring. This profile is derived by partitioning the 3D voxelized concentration map into 128 radial bins centered around the particle’s center. The concentrations within each ring are summed and divided by the number of voxels in that ring, resulting in the radial molar density profile. The impact of the microspheres packing on the average porosity profile is evident in Figure 7c. Dividing the radial molar density profile by the radial porosity profile produces the concentration profile shown in Figure 7d, which aligns well with both the average pore concentration profile and the analytical solution.

### 3.2 Reaction and Diffusion Coupled with Heat Transport

#### 3.2.1 Effectiveness Curve

In addition to the Thiele modulus (φ), the effectiveness factor (η) has a dependency on two other important dimensionless factors in the reaction-diffusion coupled with heat transfer problem: the Arrhenius Number (γ), which quantifies the sensitivity of the reaction constant to temperature, and the Prater Number (β), which is the ratio of the heat production rate and the heat removal rate due to conduction. β provides insight into the maximum possible dimensionless temperature increase within the catalyst particle.
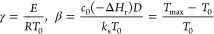
30

By solving the system of equations for the mass and heat balance, the concentration and temperature values inside pore bodies are obtained. Using these values, the effectiveness factor can be calculated as
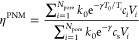
31

**Figure 8:**
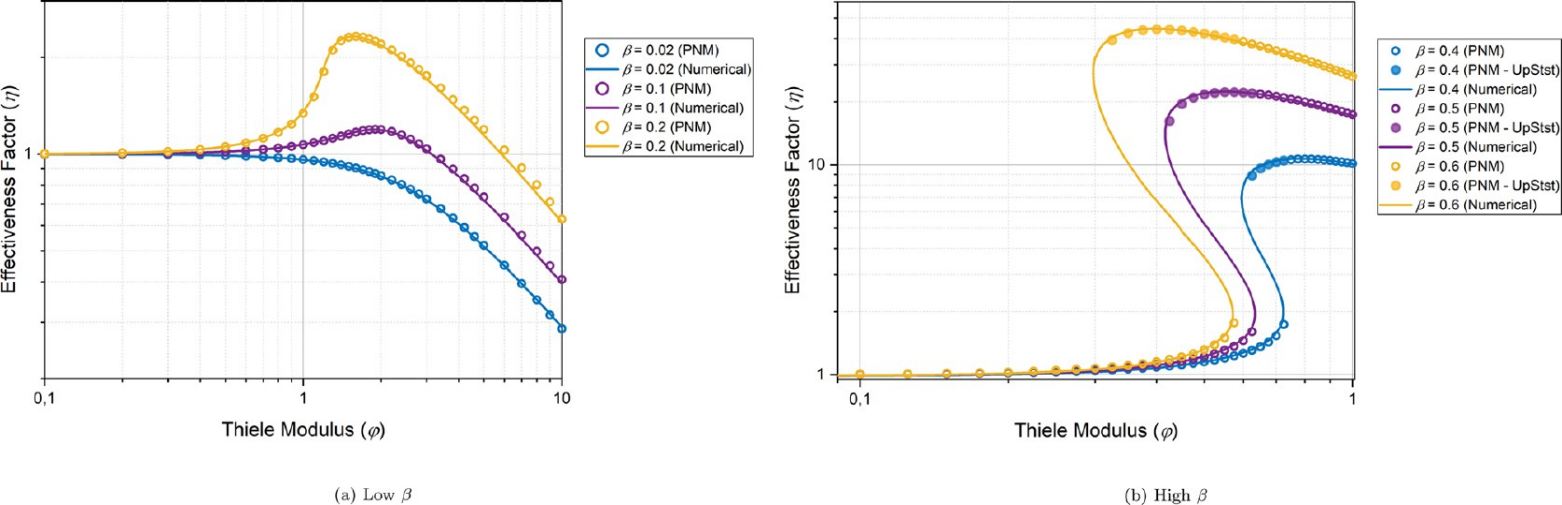
Effectiveness factor (η) curve for various β and φ values, with γ = 20. Solid symbols represent the ignited state values of the PNM solution. The filled circles represent the upper steady states obtained by setting different initial conditions.

For various Thiele moduli (φ), and Prater numbers (β), the steady-state effectiveness factor (η) is calculated and plotted in Figure 8 for γ = 20. The effectiveness factors are compared to the numerical solution provided by Weisz and Hicks.^9^ Weisz and Hicks solved the steady-state reaction-diffusion coupled with heat transport for a homogeneous spherical particle.

It can be observed from Figure 8a that with an increase in β, the effectiveness rises to values that exceed 1, and this raise beyond 1 becomes even more pronounced for higher β values. The reason behind this phenomenon is that at higher β values, the heat of reaction is high, leading to an increase in the reaction rate constant and, consequently, the reaction rate. This figure demonstrates that the proposed PNM’s are capable of accurately capturing this effect in good agreement with the numerical solution. However, the correspondence at high β and φ is weaker. This discrepancy arises from the fact that at high β and φ values, the temperature and concentration gradients at the particle surface become so steep that the currently chosen resolution of the PNM struggles to handle. Using more microspheres will alleviate the problem. Nevertheless, for such steep gradients, it can be assumed that the reaction occurs primarily on the surface of the particle, meaning that an intraparticle model is of limited value.

#### 3.2.2 Multiple Steady States

For the more exothermic reactions (high β), at higher values of φ, there are two distinct values for the effectiveness factor. This phenomenon occurs due to the interdependence of the reaction rate and temperature, as described by Arrhenius coupling. Consequently, multiple steady states are observed, with two stable points (upper and lower steady states) and one meta-stable point for specific β and φ values.^9,55^ The upper and lower steady states represent the ignited and extinguished states, respectively. Depending on the initial conditions, the solution will converge to one of the two steady states.

In Figure 8b, the filled circles represent the upper steady state. The upper steady states are obtained by setting the initial dimensionless temperature and concentration values at 1 + β and 1, respectively. Conversely, the lower steady states are obtained by setting the initial dimensionless temperature and concentration values to 1.

**Figure 9:**
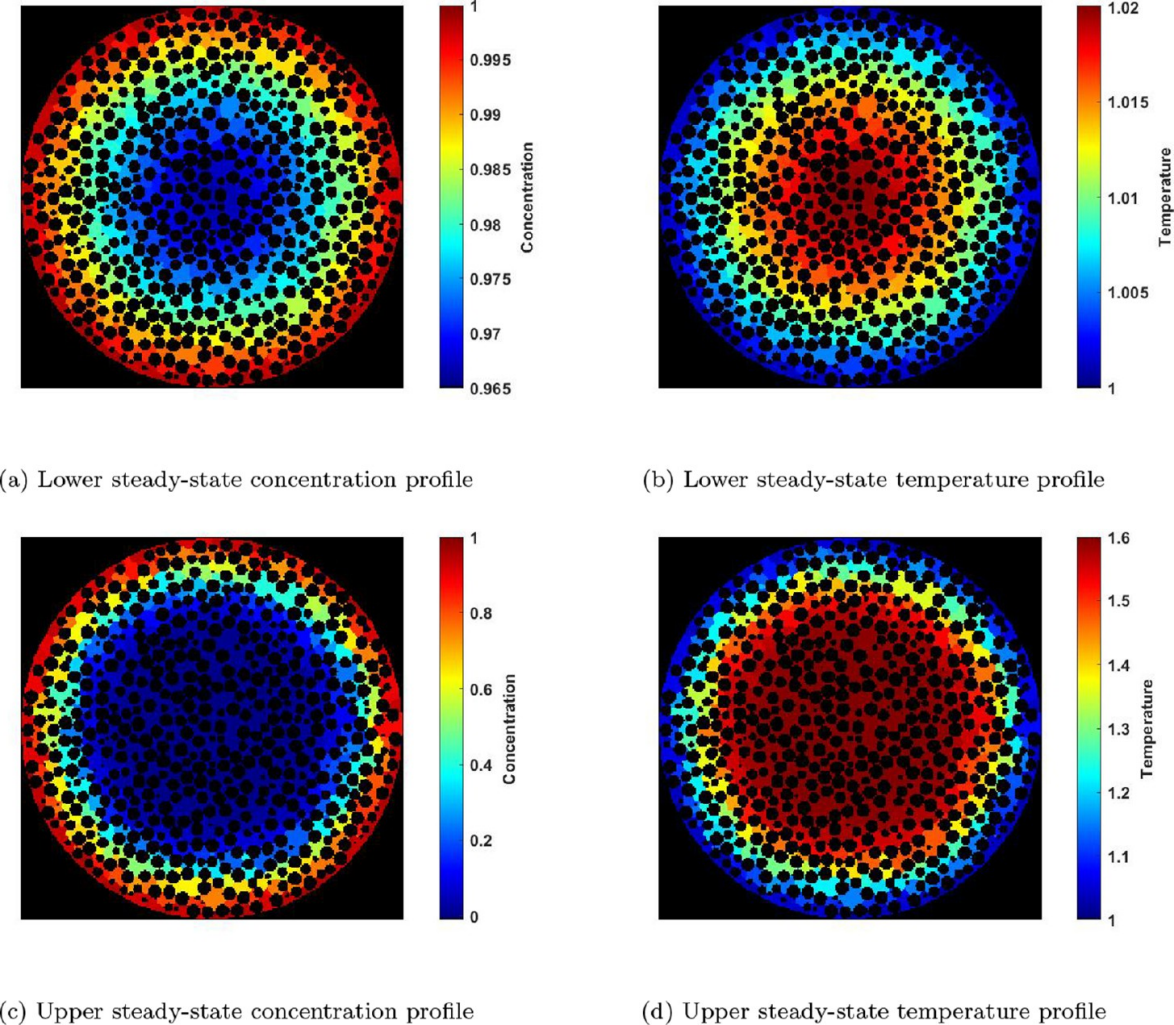
Steady-state concentration and temperature contour plots in the middle slice of the 10k particle at the lower and upper states for φ = 0.4 and β = 0.6.

**Figure 10:**
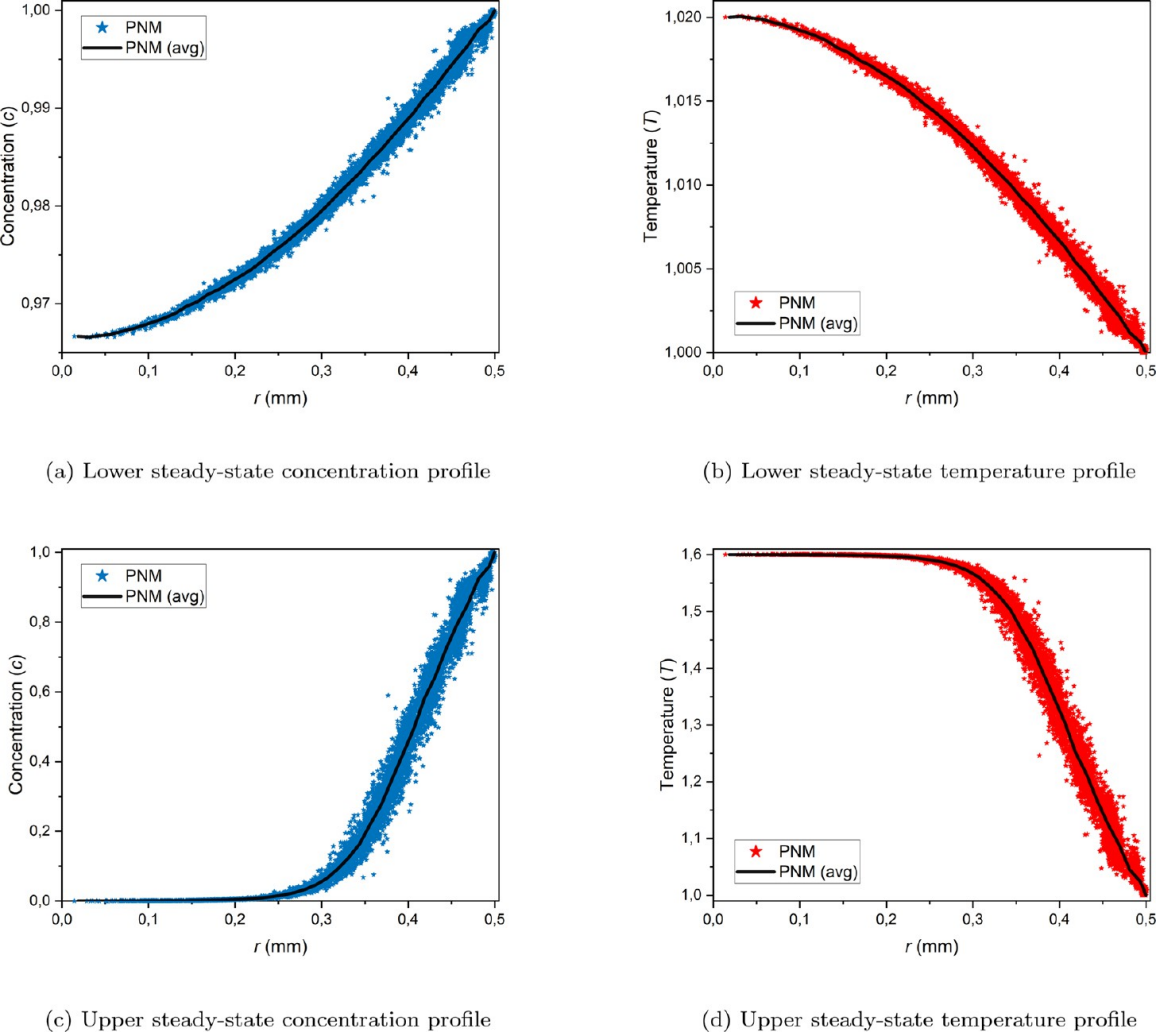
Radial steady-state concentration and temperature profiles for the 10 k particle at the lower and upper states for φ = 0.4 and β = 0.6.

Figure 9 displays the concentration and temperature maps in the central slice of the particle at the two steady states for φ = 0.4 and β = 0.6. It is evident from Figure 9 that, for the lower steady state, the variations in the concentration and temperature maps are small. However, for the upper steady state, the variations are much greater. The dimensionless temperature in the center of the particle is much higher (≈1.6), while the dimensionless concentration is zero because most of the reactant is depleted due to the high temperature and consequently, a high reaction rate. This abrupt increase in the temperature inside the particles is also termed as the ignition of the particle. To gain a deeper understanding of the concentration and temperature gradients for the lower and upper steady states, the radial concentration and temperature profiles are plotted in Figure 10. It can be seen that the gradients are much steeper for the upper steady state.

For the case of φ = 0.4 and β = 0.6, the effectiveness values are calculated for both steady states and compared to the ones from the numerical-numerical solution of Weisz and Hicks in Table 2. The comparison of these values demonstrates the ability of the PNM to accurately capture the multiple steady states, even for highly exothermic reactions.

**Table 2:** Effectiveness Values for the Lower and Upper Steady States for φ = 0.4 and β = 0.6

	PNM	Weisz and Hicks	relative error (%)
η^lower–SS^	1.154	1.158	0.35
η^upper–SS^	44.23	44.56	0.75

### 3.3 Nonuniform Boundary Condition

**Figure 11:**
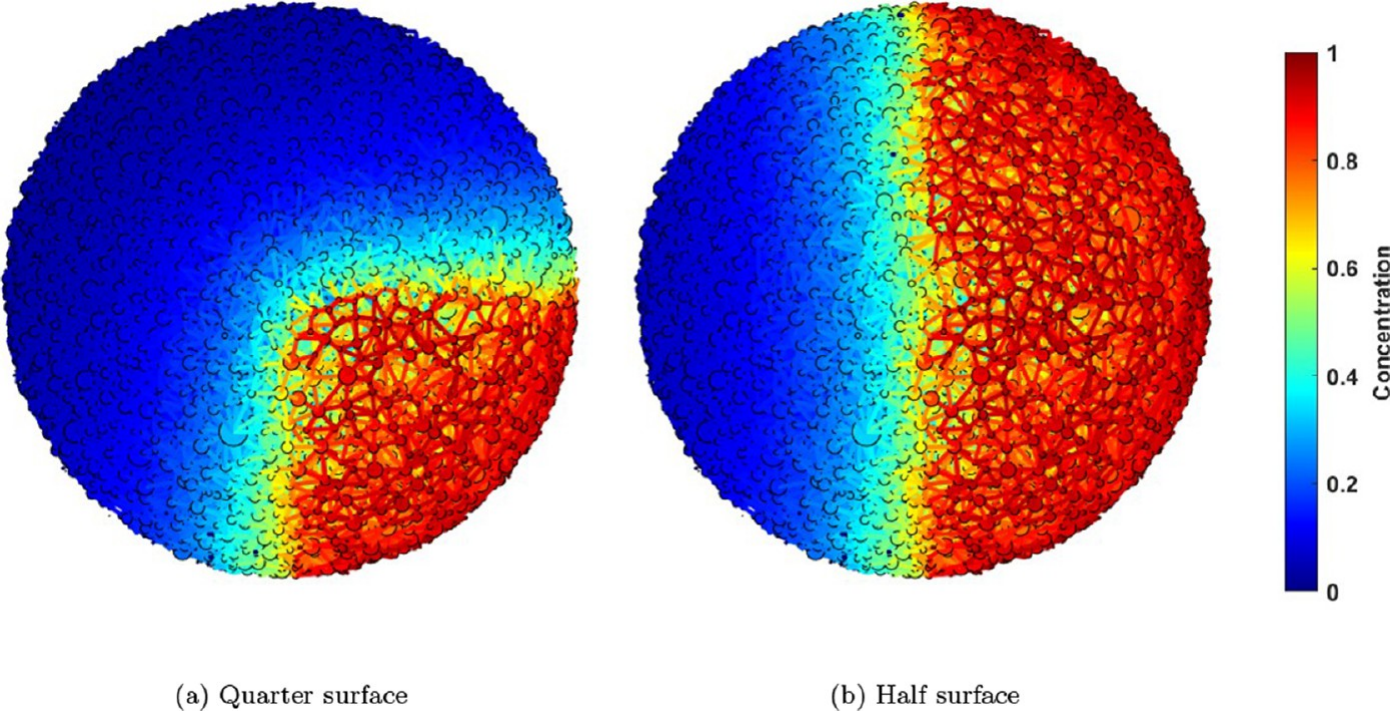
Schematic of the partially exposed PNM’s. To visualize the 3D particle-scale PNM, we adapted the Matlab code developed by Rabbani et al.^66^

The particles inside a packed bed experience nonuniform boundary conditions due to the flow distribution, particularly in slender packed beds involving multiphase (i.e., gas–liquid) operations.^14^ The developed particle-scale PNM is able to handle those nonuniform boundary conditions using the defined surface throats. In this section, as an example, two partially exposed particles are considered; one only half and the other one only a quarter of the particle surface is exposed to the environment with concentration and temperature equal to 1, and the rest of the surface is insulated. Figure 11 shows a schematic of the two partially exposed PNM’s.

**Figure 12:**
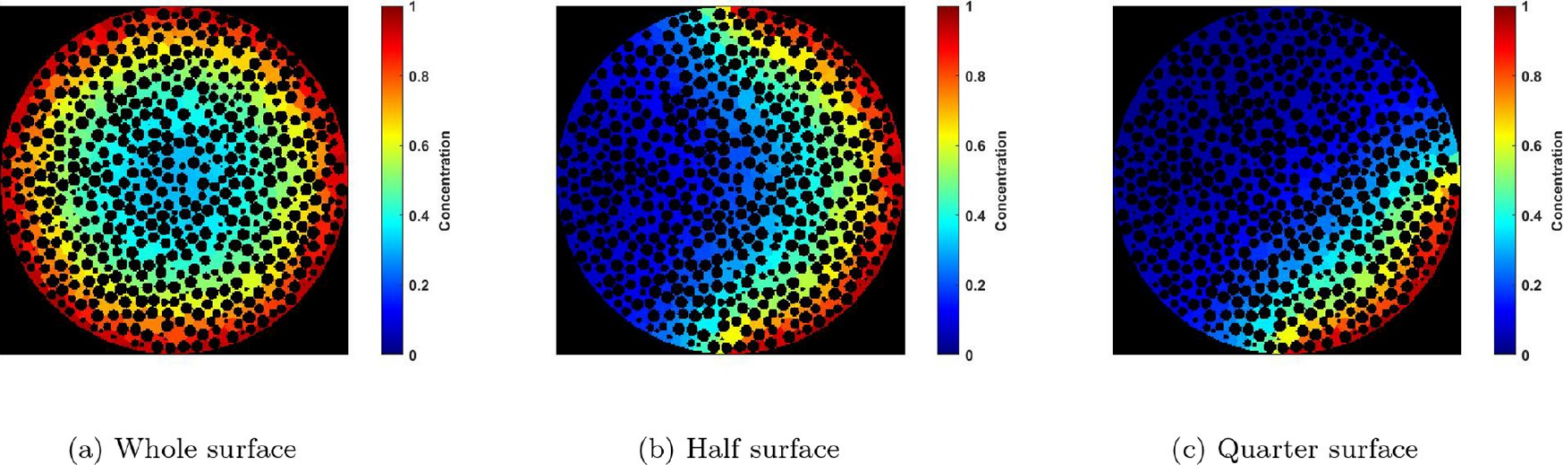
Voxelated steady-state concentration map from the middle slice of the 10k particle obtained from the reaction-diffusion inside the PNM at φ = 3 when a nonuniform boundary condition is imposed; (a) whole, (b) a half, and (c) a quarter of the surface is imposed to a boundary concentration equal to 1. The rest of the surface is insulated. The effectiveness factor (η) for each case is (a) 0.675, (b) 0.414, and (c) 0.247.

For the partially exposed PNM’s, the reaction and diffusion systems with and without heat transport is applied and the results are compared to the ones from the fully exposed PNM. For instance, Figure 12 shows the steady-state voxelated concentration maps for the reaction-diffusion inside the particle at φ = 3. It is evident that the nonuniformity of the boundary condition effects the concentration map. For instance, in the case of a half-exposed particle, crescent-shaped concentration iso-surfaces extend from the surface in contact with the environment to the isolated surface.

**Figure 13:**
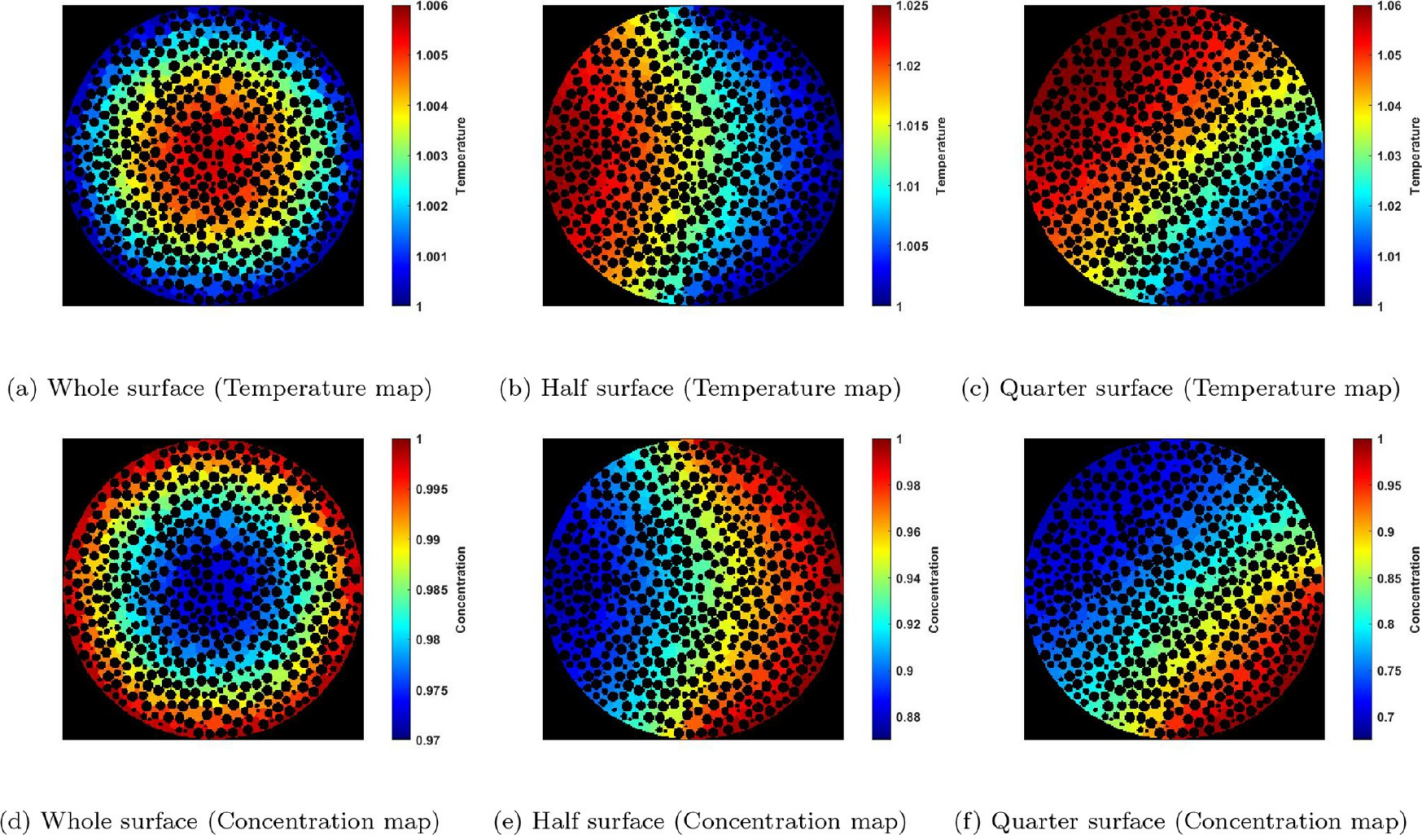
Voxelated steady-state concentration and temperature maps from the middle slice of the 10k particle obtained from PNM at φ = 0.4 and β = 0.2 when a nonuniform boundary condition is imposed; (a,d) whole, (b,e) a half, and (c,f) a quarter of the surface is imposed to a boundary concentration and temperature equal to 1. The rest of the surface is insulated.

For the reaction-diffusion coupled with heat transfer, this becomes even more interesting. Figure 13 displays the concentration and temperature maps for φ = 0.4 and β = 0.2 in the case of partially exposed PNM’s. It can be seen from the temperature map that, in the partially exposed PNM’s, there is a significant temperature increase in regions with lower exposure to the external environment. This local temperature increase, caused by flow mal-distribution within the packed bed, is at the core of hot-spot formation. The elevated temperature increases the reaction rate, resulting in concentration and temperature profiles that exhibit an inverse relationship.

In this model, the reason for this temperature increase is due to the assumption of an insulated surface in the model. In reality, some heat transfer is possible from this surface, but it may be limited by external transfer limitations. The Biot number (Bi = *hR*_p_/λ) is a dimensionless number used to characterize the relative significance of internal heat conduction within a particle in contrast to the external heat convection from its surface to the surrounding environment. In many cases, particularly when dealing with highly conductive solid materials (e.g., metal catalysts) and relatively low gas flow rates, the Biot number can be significantly less than 1. This could mean that the internal generation of heat due to reaction in some locations of the bed cannot effectively be transported by the convective flow through the particle bed.

**Figure 14:**
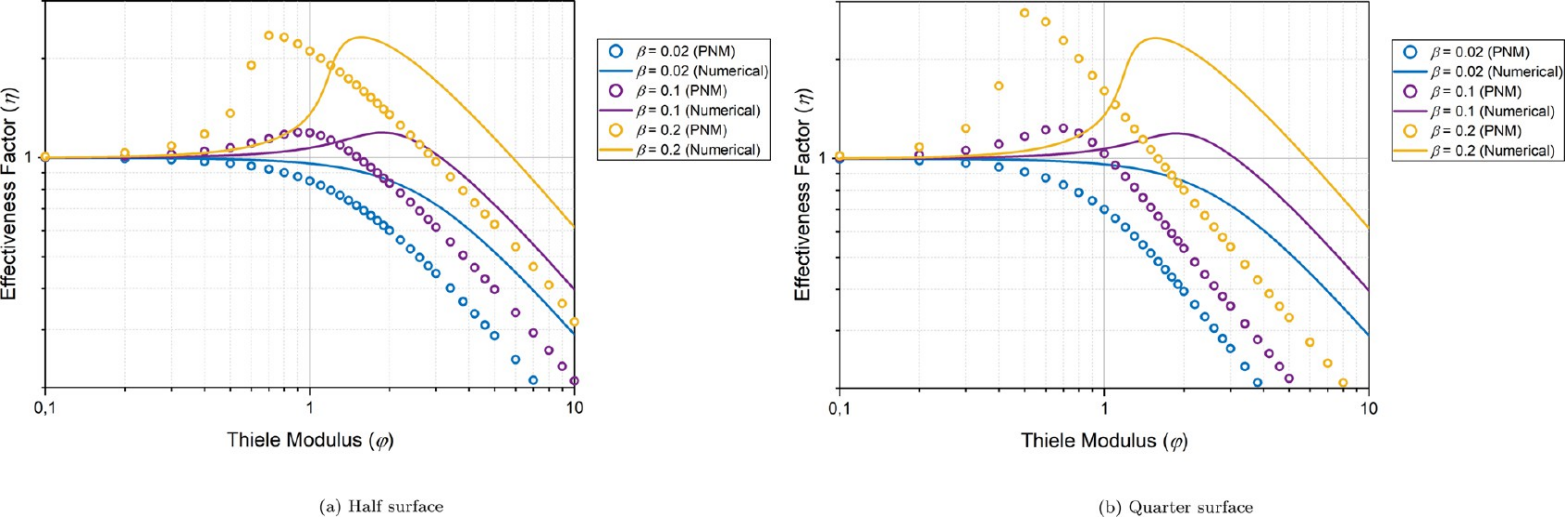
Effectiveness factor (η) curve for various β and φ values for the partially exposed particles and comparison to the numerical solution of Weisz and Hicks for the fully exposed homogeneous spherical particle.

For the partially exposed PNM’s, the effectiveness factor at various φ and β values are calculated and the effectiveness profiles are compared to the numerical solution of Weisz and Hicks for a fully exposed homogeneous spherical particle (Figure 14). It is noteworthy that, for the partially exposed particles, there is a shift in the effectiveness curve where the increase in effectiveness due to temperature effects occurs at lower φ values. This is logical because the generated heat is not efficiently removed from the nonexposed region by the environment. The shift toward lower φ values is particularly pronounced in the case of quarter surface exposure, where the contact area with the environment is reduced. Additionally, in the case of quarter surface exposure, there is also an increase in the maximum value of the effectiveness factor due to the heat accumulation when the β value is sufficiently high (β = 0.6).

**Figure 15:**
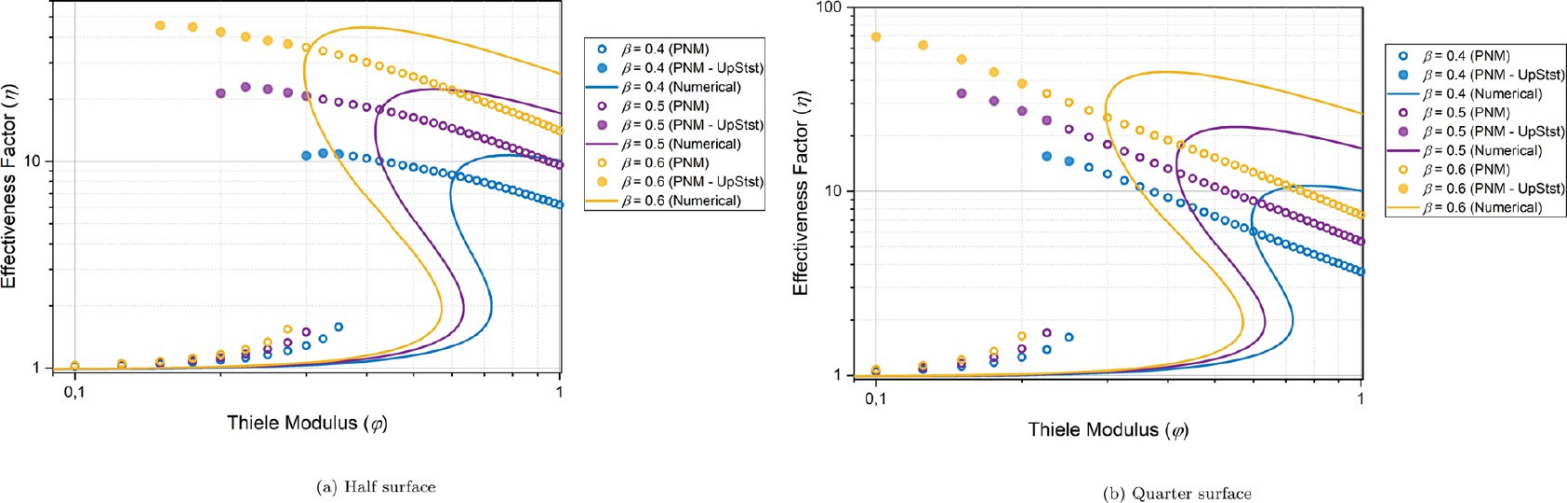
Effectiveness factor (η) curve for various β and φ values for the partially exposed particles and comparison to the numerical solution of Weisz and Hicks for the fully exposed homogeneous spherical particle. The filled circles represent the upper steady states obtained by setting different initial conditions.

It was demonstrated in Section 3.2.2 that highly exothermic reactions inside a particle can lead to the existence of two stable steady states. In this section, we explore this phenomenon in the context of partially exposed particles. Figure 15 illustrates the effectiveness curves for high β values. Similar behavior is observed here, where the partial exposure of the particle causes the effectiveness curve to shift toward lower φ values. For instance, at β = 0.4, no upper steady state is observed at φ = 0.3 for a fully exposed homogeneous spherical particle. However, for half-surface exposure, the upper steady state exists at φ = 0.3. In the case of quarter-surface exposure, the sudden increase in the effectiveness (ignition) occurs at even lower φ values.

## 4 Conclusions

In conclusion, this study introduces a novel 3D PNM designed to simulate reaction-diffusion processes, incorporating the more intricate scenario of reaction-diffusion coupled with heat transfer within spherical porous catalyst particles. The model effectively represents the 3D shape and intricate porosity and tortuosity inherent in such particles in a computationally efficient manner by utilizing an agglomeration of ten thousand microspheres. The particle-scale PNM is calibrated by fitting the effectiveness curve at various Thiele values from the PNM to those obtained from the analytical solution. The validation of the model is confirmed through a comparison of the radial concentration profiles derived from the PNM and the analytical solution. Additionally, the comparison of effectiveness curves for reaction-diffusion coupled with heat transfer from the PNM to those from the numerical solution further validates the model’s applicability for these complex processes.

Notably, our particle-scale PNM offers the capability to apply realistic 3D boundary conditions directly on the particle surface. This feature holds substantial importance, especially in the context of slender packed bed reactors involving multiphase operations, where accurate representation of the particle’s surface conditions is crucial. Achieving this capability involves the definition and characterization of surface throats and pores within our model. In essence, our particle-scale PNM goes beyond traditional models by allowing for a more realistic and refined simulation of reaction-diffusion processes within spherical porous catalyst particles.

Another intriguing area for exploration in this field involves introducing fluid flow into the porous particle and coupling the flow hydrodynamics with mass and heat transport phenomena. This investigation aims to comprehend the effects of fluid flow on transport phenomena within the porous structure of catalyst pellets using the PNM. It is important to note that fluid flow outside the catalyst particle can influence external transfer limitations. Therefore, a thorough investigation of the potential external transfer limitations in relation to the Biot number and how these factors may impact the PNM model is another crucial avenue for future studies.

Furthermore, exploring different particle shapes and geometries, such as cylindrical, spherocylindrical, cubical, and oval particles, holds promise. Each of these particles can be simulated as composed of thousands of microspheres, allowing for the replication of complex porous catalyst structures. The number of microspheres composing a porous pellet is another intriguing parameter deserving of investigation. Analyzing how changes in the number of microspheres impact transport phenomena within the porous pellet, how these results deviate from theoretical models, and their effects on effective diffusivity inside the catalyst particle all present promising research opportunities in this field.

The developed particle-scale PNM is a fast particle model capable of conducting combined reaction-diffusion with and without heat transfer in a matter of seconds, without requiring high computational resources. Consequently, it can be employed to model catalyst particles in packed bed reactors, where hundreds to thousands of catalyst particles exist, coupling them with reactor-scale transport phenomena. However, in this work, we have made some assumptions such as considering volumetric reactions and heat conduction through the pore network. Incorporating considerations such as surface reactions and a more detailed heat transfer model would be valuable for future research efforts aimed at enhancing the accuracy of our model while maintaining computational efficiency.
